# Complete Structure of the Enterococcal Polysaccharide Antigen (EPA) of Vancomycin-Resistant Enterococcus faecalis V583 Reveals that EPA Decorations Are Teichoic Acids Covalently Linked to a Rhamnopolysaccharide Backbone

**DOI:** 10.1128/mBio.00277-20

**Published:** 2020-04-28

**Authors:** Yann Guerardel, Irina Sadovskaya, Emmanuel Maes, Sylviane Furlan, Marie-Pierre Chapot-Chartier, Stéphane Mesnage, Lionel Rigottier-Gois, Pascale Serror

**Affiliations:** a Univ. Lille, CNRS, UMR 8576—UGSF—Unité de Glycobiologie Structurale et Fonctionnelle, Lille, France; b Univ. Littoral Côte d’Opale, UMR 1158 BioEcoAgro, TERRA Viollette, USC Anses, INRAe, Univ. Lille, Univ. Artois, Univ. Picardie Jules Verne, Univ. Liège, Yncréa, Boulogne-sur-Mer, France; c Université Paris-Saclay, INRAE, AgroParisTech, Micalis Institute, Jouy-en-Josas, France; d University of Sheffield, Department of Molecular Biology and Biotechnology, Sheffield, United Kingdom; University of Minnesota Medical School

**Keywords:** *E. faecalis*, teichoic acids, cell wall polysaccharide, enterococcal polysaccharide antigen, *Enterococcus faecalis*, rhamnan

## Abstract

All enterococci produce a complex polysaccharide called the enterococcal polysaccharide antigen (EPA). This polymer is required for normal cell growth and division and for resistance to cephalosporins and plays a critical role in host-pathogen interaction. The EPA contributes to host colonization and is essential for virulence, conferring resistance to phagocytosis during the infection. Recent studies revealed that the “decorations” of the EPA polymer, encoded by genetic loci that are variable between isolates, underpin the biological activity of this surface polysaccharide. In this work, we investigated the structure of the EPA polymer produced by the high-risk enterococcal clonal complex Enterococcus faecalis V583. We analyzed purified EPA from the wild-type strain and a mutant lacking decorations and elucidated the structure of the EPA backbone and decorations. We showed that the rhamnan backbone of EPA is composed of a hexasaccharide repeat unit of C2- and C3-linked rhamnan chains, partially substituted in the C3 position by α-glucose (α-Glc) and in the C2 position by β-*N*-acetylglucosamine (β-GlcNAc). The so-called “EPA decorations” consist of phosphopolysaccharide chains corresponding to teichoic acids covalently bound to the rhamnan backbone. The elucidation of the complete EPA structure allowed us to propose a biosynthetic pathway, a first essential step toward the design of antimicrobials targeting the synthesis of this virulence factor.

## Abstract (precis)

Enterococci are opportunistic pathogens responsible for hospital- and community-acquired infections. All enterococci produce a surface polysaccharide called EPA (*e*nterococcal *p*olysaccharide *a*ntigen) required for biofilm formation, antibiotic resistance, and pathogenesis. Despite the critical role of EPA in cell growth and division and as a major virulence factor, no information is available on its structure. Here, we report the complete structure of the EPA polymer produced by the model strain E. faecalis V583. We describe the structure of the EPA backbone, made of a rhamnan hexasaccharide substituted by Glc and GlcNAc residues, and show that teichoic acids are covalently bound to this rhamnan chain, forming the so-called “EPA decorations” essential for host colonization and pathogenesis. This report represents a key step in efforts to identify the structural properties of EPA that are essential for its biological activity and to identify novel targets to develop preventive and therapeutic approaches against enterococci.

## INTRODUCTION

Enterococci are commensal bacteria colonizing the gastrointestinal tract of virtually all animals, including humans. While these organisms are usually harmless, they can cause a wide range of infections in immunocompromised patients or following dysbiosis caused by an antibiotic treatment (1). Enterococcus faecalis and Enterococcus faecium have emerged as common opportunistic pathogens, E. faecalis being the species most frequently isolated from community- and hospital-acquired infections (2–4). Several components of the E. faecalis cell envelope have been shown to play important roles during host-pathogen interactions (for reviews, see references 5 and 6). These include lipoteichoic teichoic acids (LTAs); the capsular polysaccharide (CPS), which is variable and nonubiquitous; wall teichoic acids (WTAs); and the enterococcal polysaccharide antigen (EPA). The LTAs are anchored to the membrane by a glycolipid moiety and consist of a glycerol phosphate polymer with a kojibiose substitution and a d-alanylation decoration (7–9). All of the other polymers (EPA, WTA, and CPS) are covalently bound to peptidoglycan, the essential and major component of the cell envelope. CPS is a diheteroglycan of glucose (Glc) and galactose (Gal) involved in resistance to phagocytosis (10–12). E. faecalis WTAs have a complex structure; the repeating unit of the WTA from E. faecalis strain 12030 is composed of d-glucose (Glc), d-galactose (Gal), 2-acetamido-2-deoxy-d-galactose, 2-acetamido-2-deoxy-d-glucose, d-ribitol (Rbo), and phosphate (13), whereas E. faecalis strain V583 harbors two types of WTA composed of repeating units of Rbo-containing trisaccharides or of repeating units of Rbo-containing tetrasaccharides (14). Both LTAs and WTAs confer an anionic surface charge and play roles in resistance to host phagocytosis and in inflammation (15). EPA from numerous strains contains rhamnose (Rha), *N*-acetylgalactosamine (GalNAc), Gal, and *N*-acetylglucosamine (GlcNAc) (16–18). This ubiquitous polymer plays a role in multiple processes such as biofilm formation, adhesion to the intestinal mucus and translocation through epithelial cells, resistance to phagocytosis and to antimicrobials, virulence in various infection models, and phage infection (16, 17, 19–25). Despite the pivotal role of EPA in enterococci, the structure and properties of this polymer required for its biological activities remain largely unknown. We and others have shown that the gene products involved in EPA biosynthesis are encoded by a 40.6-kb locus composed of two genetic loci: a conserved cluster, consisting of 18 genes (*ef2198* to *ef2177* in V583) and a cluster of 10 to 20 genes (18 genes [*ef2165* to *ef2177*] in V583) presenting genetic variability featuring major differences in EPA between E. faecalis isolates (18, 19, 24, 26). These clusters are reminiscent of loci involved in biosynthesis of rhamnose-containing polysaccharides (27). The proteins encoded by these clusters include dTDP-rhamnose biosynthesis enzymes (EF2191 to EF2194), putative group 4 and group 2 glycosyltransferases, LicD-related proteins (EF2165 and EF2172), O-antigen ligase (EF2169), and glycoside hydrolase family 25 (EF2174). We discovered that EPA from E. faecalis strain V583 is required for intestinal colonization. We also identified EpaX (EF2170), a putative glycosyltransferase involved in cell wall integrity and resistance to bile salts (18), corresponding to which homologs in another strain have similar roles (17, 19, 24). The proteins involved in the synthesis of the EPA rhamnan “backbone” have been predicted to be encoded by genes in the conserved region, whereas we hypothesized that EpaX, as well as, more generally, the gene products of the variable region, plays a role in the decoration of this backbone.

In this study, we determined the complete structure of the EPA from a derivative of E. faecalis V583. We revealed that EPA consists of a rhamnan backbone to which teichoic acids (so-called “EPA decorations”) are covalently bound. Using high-resolution magic angle spinning nuclear magnetic resonance spectroscopy (HR-MAS NMR) on whole bacteria, we showed that rhamnan is buried within the cell wall and that only decorations of EPA are exposed at the bacterial surface. On the basis of the structural data presented, we propose a functional predictive model of the biosynthetic pathway of EPA.

## RESULTS

### EPA backbone and decorations are linked by phosphodiester bonds.

**FIG 1:**
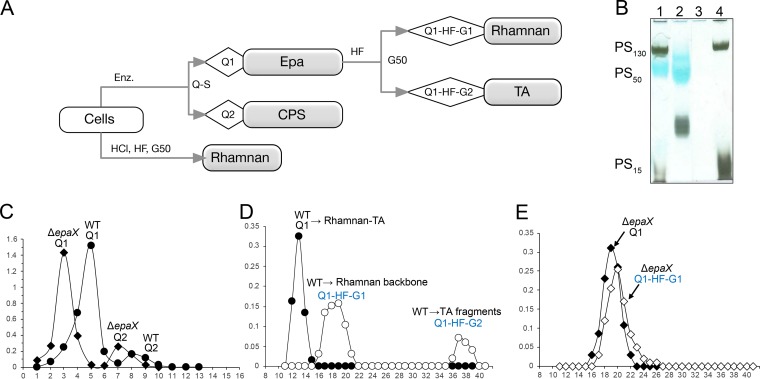
Biochemical analysis of cell wall polysaccharide (CWP) fractions in E. faecalis VE14089 WT. (A) Summary of the experimental strategy showing the presence of covalently linked rhamnan and TA in WT. On the one hand, Epa (Q1) was purified exclusively from the WT strain by enzymatic degradation. Epa could be further decomposed into rhamnan (Q1-HF-G1) and TA (Q1-HF-G2) fragments by HF treatment. The Q1 fraction extracted from the Δ*epa*X mutant strain contained only rhamnan. A CPS fraction (Q2) was identified in both strains. On the other hand, structurally identical rhamnan fractions were extracted from the WT and Δ*epa*X mutant strains by treatment with HCl, followed by HF. Components in gray boxes were subjected to detailed NMR structural analysis and methylation analysis. Enz., enzymatic degradation with lysozyme, mutanolysin, DNase, RNase, and proteinase K; G50, fractionation on a Sephadex G50 column; Q-S, ion-exchange chromatography on a Q-Sepharose fast flow column. (B) SDS-PAGE with alcian blue/silver nitrate staining of crude cell wall enzymatic digests and fractions purified by anion-exchange chromatography. Lane 1, WT enzymatic CWP preparation; lane 2, WT enzymatic CWP preparation, fraction WT-Q1; lane 3, Δ*epa*X mutant enzymatic CWP preparation, fraction Δ*epa*X-Q1; lane 4, Δ*epa*X mutant enzymatic CWP preparation. Polysaccharides PS_130_, PS_50_, and PS_15_ are named according to the size (in kilodaltons) determined by Hancock and Gilmore (28). (C) Elution profiles of enzymatic CWP preparations from the WT and the Δ*epaX* mutant on an anion exchange column. (D and E) Chromatographic profiles after gel filtration on a Sephadex G-50 column (2.6 by 90 cm) of enzymatic CWP preparations from the WT (D) and from the Δ*epa*X mutant (E) before (full circles and full diamonds) and after (empty circles and empty diamonds) treatment with 48% HF. Fractions are named as described for panel B. In panels C to E, x-axis numbers represent fraction numbers and y-axis numbers represent total sugar assay absorption at 485 nm (Abs).

Electrophoretic mobility experiments and staining with the cationic dye alcian blue showed that EPA decorations confer a negative charge to the enterococcal cell surface (14, 16, 18, 24). We have previously shown that rhamnopolysaccharide Epa of E. faecalis V583 and OG1RF is not detected in mutants deleted for the *epaX* gene by the cationic dye alcian blue whereas it appears as a blue band in the wild type (WT) (18, 24). In order to further characterize the Epa molecule, we used a method preserving acid-labile bonds and allowing the characterization of carbohydrates in their native form. We therefore used mutanolysin to cleave the peptidoglycan to which E. faecalis cell wall polymers are covalently bound, as described previously (18) (Fig. 1A). In agreement with previous studies (18, 28), PAGE analyses of the crude enzymatic extract of the WT strain confirmed the presence of three polymers originally described as PS_130_, PS_50_, and PS_15_ (Fig. 1B, lane 1). The band corresponding to EPA (PS_50_) was not detected in the Δ*epaX* extract (Fig. 1B, lane 4). Further fractionation of the enzymatic extracts on an anion exchange Q-Sepharose column gave two fractions (Q1 and Q2) for both strains (Fig. 1C). PAGE analysis indicated that fraction WT-Q1 (Fig. 1B, lane 2) corresponded to EPA. Since the second band from WT-Q1 was not present in the original extract, we assumed that it corresponded to a product of partial degradation of EPA. In agreement with previous studies (18, 24), fraction Δ*epaX*-Q1 was eluted earlier on the ion-exchange column (Fig. 1C) and was not stained by alcian blue (Fig. 1B), indicating that it was not negatively charged or was much less negatively charged. The two fractions eluting later (WT-Q2 and Δ*epaX*-Q2) were identified as the capsular polysaccharide (see below).

Fractions WT-Q1 and Δ*epaX*-Q1 were further analyzed. While both WT-Q1 and Δ*epaX*-Q1 eluted as single fractions (Fig. 1D and E) on a Sephadex G-50 column, treatment of fraction WT-Q1 with aqueous hydrogen fluoride (HF) yielded two fractions that could be separated by gel filtration: high-molecular-weight WT-Q1-HF-G1 and low-molecular-weight WT-Q1-HF-G2 (Fig. 1D, empty circles). In contrast, treatment of fraction Δ*epaX*-Q1 with HF did not affect its elution profile on the Sephadex G-50 column (Fig. 1E). Both the WT-Q1-HF-G1 and Δ*epaX*-Q1-HF-G1 high-molecular-weight fractions contained l-Rha, d-Glc, and d-GlcNAc in an approximate ratio of 1:0.3:0.3. Methylation analyses indicated that the two fractions were identical and made of 2-, 3-, and 2,3-linked Rha, terminal Glc, and terminal GlcNAc residues. WT-Q1-HF-G2 contained Rha, Rbo, Glc, and GalNAc. In line with the results of gas chromatography-mass spectrometry (GC-MS) analyses, the ^1^H NMR spectra of WT-Q1-HF-G1 and Δ*epaX*-Q1-HF-G1 were virtually identical (see Fig. S1 in the supplemental material).

10.1128/mBio.00277-20.1 FIG S1 (A and B) ^1^H NMR spectra of the rhamnan backbone of (A) the cell wall polysaccharides from the E. faecalis WT strain and (B) the Δ*epa*X mutant purified by sequential mutanolysin digestion and HF hydrolysis (Fig. 1A). (C and D) ^1^H NMR spectra of rhamnan from (C) WT and (D) the Δ*epa*X mutant *epa*X by acid hydrolysis (Fig. 1A). Download FIG S1, PDF file, 0.2 MB. Copyright © 2020 Guerardel et al. 2020 Guerardel et al. This content is distributed under the terms of the Creative Commons Attribution 4.0 International license.

Together, these data indicated that WT-Q1 contained a polysaccharide molecule from which short fragments could be cleaved off with HF and that it therefore contained fragments attached to the main “backbone” with phosphodiester bonds. These fragments were absent for the Δ*epaX*-Q1 preparation, indicating that Δ*epaX*-Q1 was composed of the backbone only, leading to the conclusion that the EPA decorations conferring a negative charge to the enterococcal cell surface were linked to the EPA backbone via phosphodiester bonds.

### EPA is built on a ramified rhamnan backbone.

**FIG 2:**
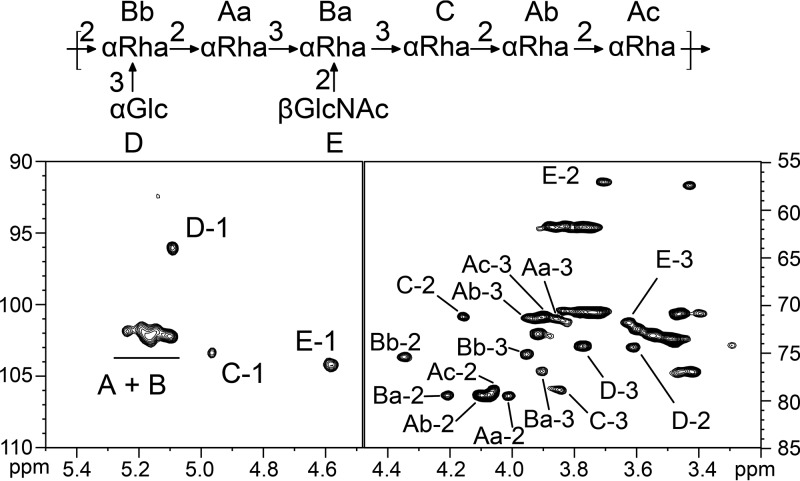
NMR structural analysis of the rhamnan backbone of EPA. The figure presents 2D HSQC NMR and structural elements identified in the rhamnan backbone of E. faecalis VE14089 WT. The rhamnan backbone was extracted and purified from the WT strain by sequential treatment with HCl and HF, as depicted in Fig. 1A (bottom pathway). The *x*- and *y*-axis numbers in the lower panels represent ppm.

To elucidate the detailed structure of the Epa backbone by two-dimensional (2D) NMR analysis, large amounts of backbone fraction were prepared from the wild-type and Δ*epaX* strains, using hot acid extraction instead of enzymatic digestion, followed by HF treatment (Fig. 1A) (29). The monosaccharide compositions of these preparations were identical to those of the enzymatic ones, WT-Q1-HF-G1 and ΔepaX-Q1–HF-G1. The ^1^H NMR spectra of the two preparations from the WT strain and the Δ*epaX* mutant were also virtually identical (Fig. S1). NMR analysis revealed a complex pattern of anomeric signals grouped in four main regions (Fig. 2). The spin systems of individual residues in each group of signals were established by a combination of 2D NMR experiments, including ^1^H/^1^H correlation spectroscopy (COSY), total correlation spectroscopy (TOCSY), nuclear Overhauser effect spectroscopy (NOESY), rotating-frame Overhauser effect spectroscopy (ROESY), and ^1^H/^13^C heteronuclear single quantum coherence (HSQC) spectroscopy (Table 1). A crowded region of anomeric protons at 5.105 to 5.240/101.0 to 102.0 ppm exclusively contained αRha*p* residues that were either monosubstituted in the C2 position (2-αRha; labeled as “A” residues in Fig. 2) or disubstituted in the C2 and C3 positions (2,3-αRha; labeled as “B” residues in Fig. 2). Among the 2-αRha residues, two were interconnected 2-αRha*p* residues that differed only in their relative positioning within the polysaccharide chain. This hypothesis was later confirmed by NOESY. Among the 2,3-αRha*p* residues, two distinct spin systems could be clearly distinguished corresponding to “Ba” and “Bb” residues. In addition, the anomeric signal at 4.966/103.24 ppm was associated with another type of αRha*p* residue monosubstituted in the C3 position (3-αRha; labeled as “C” in Fig. 2). Finally, anomeric signals at 5.089/95.95 ppm (“D”) and at 4.582/104.05 ppm (“E”) were associated with unsubstituted α-Glc*p* (t-αGlc) and βGlcNAc*p* (t-βGlcNAc) residues, respectively. The sequence of the polysaccharide was established using ^1^H-^1^H ROESY and ^1^H-^13^C heteronuclear multiple-bond connectivity (HMBC) maps, as shown in Table 2. Most notably, 2,3-αRha “Bb” residue was substituted in C2 position by 2-αRha “Ac” and in C3 by t-αGlc residue “D.” NOESY contact showed that the 2,3-αRha “Bb” residue was substituting the 2-αRha “Aa” residue in C2 position, but no clear HMBC contact could be observed between these two residues due to region overcrowding. In addition, clear NOESY and HMBC contacts showed that a 2-αRha “Aa” residue was linked to a 2,3-αRha “Ba” residue and that the “Ba” residue was linked to a 3-αRha “C” residue, both in the C3 position. The 2,3-αRha “Ba” residue was further substituted by a t-βGlcNAc “E” residue at the C2 position. Finally, intense NOESY contacts strongly suggested the presence of a “C”-2“Ab”-2-“Ac”-2-linked stretch of Rha residues. Collectively, NMR and GC-MS linkage analyses revealed that the EPA backbone consists of a rhamnan chain substituted in the C3 position by αGlc and in the C2 position by βGlcNAc residues, with a proposed sequence of -[-2(αGlc1-3)αRha1-2αRha1-3(βGlcNAc1-2)αRha1-2αRha1-2αRha1-2αRha1-]- (Fig. 2) and that the EPA decorations are attached to the rhamnan backbone via phosphodiester bonds.

**TABLE 1:** ^1^H and ^13^C NMR chemical shift values for the WT rhamnan fraction liberated by HCl and HF treatment and purified by fractionation on a Sephadex G-50 column

Fraction	Residue	^1^H and ^13^C chemical shifts (ppm)						
		H1, C1	H2, C2	H3, C3	H4, C4	H5, C5	H6, C6	H6′, C6
→2)-α-Rha*p*→	Aa	5.235, 101.84	4.011, 79.47	3.861, 71.48	3.545, 73.11	3.734, 70.74	1.333, 18.04	
→2)-α-Rha*p*→	Ab	5.109, 102.16	4.106, 79.45	3.901, 71.27	3.471, 73.52	3.74–3.78, 70.5	1.28, 18.0	
→2)-α-Rha*p*→	Ac	5.159, 102.07	4.067, 79.37	3.944, 71.37	3.513, 73.49	3.74–3.78, 70.5	1.28, 18.0	
→2,3)-α-Rha*p*→	Ba	5.171, 101.82	4.210, 79.33	3.904, 76.80	3.484, 73.45	3.728, 70.65	1.282, 18.1	
→2,3)-α-Rha*p*→	Bb	5.173, 102.25	4.347, 75.35	3.953, 75.10	3.621, 71.72	3.738, 70.65	1.280, 18.01	
→3)-α-Rha*p*→	C	4.966, 103.24	4.159, 71.13	3.843, 78.7	3.558, 72.93	3.779, 70.61	1.282, 18.11	
t-α-Glc*p*→	D	5.095, 95.95	3.608, 74.29	3.772, 74.08	3.919, 72.93	3.463, 70.80	3.748, 61.72	3.782, 61.72
t-β-GlcNAc*p*→	E	4.582, 104.05	3.702, 57.21	3.628, 71.7	3.468, 73.47	3.425, 76.85	3.766, 61.66	3.872, 61.66

**TABLE 2:** Summary of the ^1^H-^1^H ROESY and ^1^H-^13^C HMBC contacts observed in the WT rhamnan fraction^*a*^

Fraction	Residue	NOE	HMBC
→2)-α-Rha*p*-(1→	Aa	Aa-H1 → Ba-H3	Aa-H1 → Ba-C3
			Ba-H3 → Aa-C1
	Ab	Ab-H1 → Ac-H2	nd
	Ac	Ac-H1 → Bb-H2	nd

→2,3)-α-Rhap-(1→	Ba	Ba-H1 → C-H3	C-H3 → Aa-C1
	Bb	Bb-H1 → Aa-H2	nd

→3)-α-Rhap-(1→	C	C-H1 → Ab-H2	C-H1 → Ab-C2

t-α-Glc*p*-(1→	D	D-H1 → Bb-H2	Bb-H3 → D-C1
		D-H1 → Bb-H3	

t-β-GlcNAc*p*-(1→	E	E-H1 → Ba-H2	E-H1 → Ba-C2
			Ba-H2 → E-C1

a NOE, nuclear Overhauser effect; HMBC, heteronuclear multiple-bond connectivity; nd, not detected.

### EPA rhamnan backbone is substituted by a teichoic-like polysaccharide.

**FIG 3:**
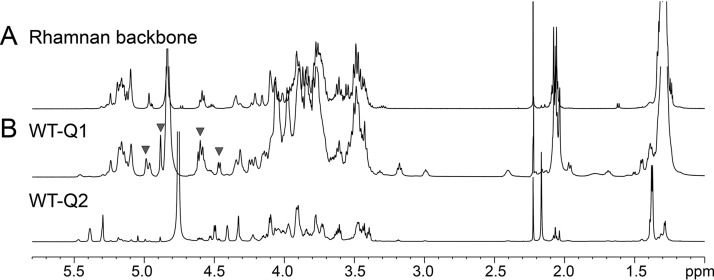
Comparison of the ^1^H NMR spectra of polysaccharide fractions purified from E. faecalis VE14089 WT. (A) Spectrum of rhamnan extracted and purified by sequential treatment with HCl and HF. (B) Spectra of WT-Q1 and WT-Q2 purified by anion-exchange chromatography following enzymatic degradation of the cell wall from E. faecalis. Fractions are named as described for panel Fig. 1A. Inverted filled triangles highlight signals that were observed for the WT-Q1 fraction (middle spectrum) but not for rhamnan (top spectrum).

**FIG 4:**
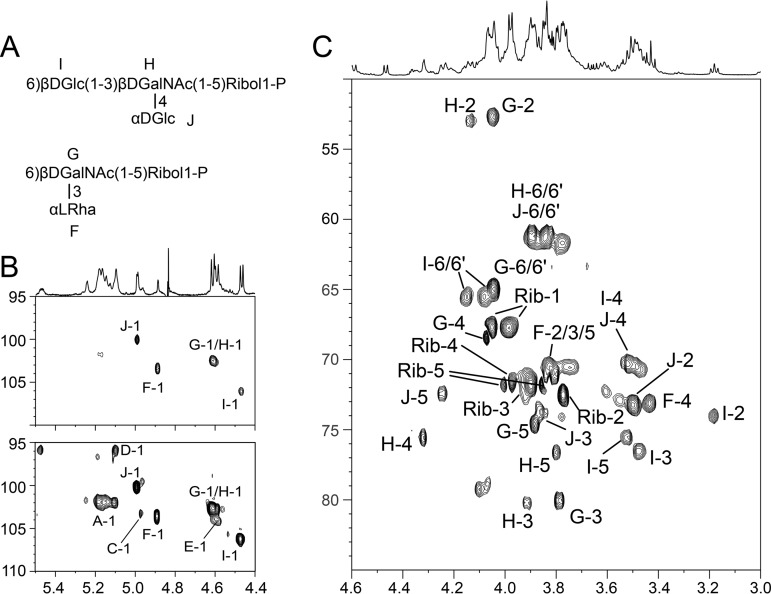
NMR analysis of the purified rhamnan-TA preparation (WT-Q1) from E. faecalis VE14089 WT. (A) Structures of the identified TA repeating units. (B) Anomeric region of the ^1^H/^13^C HSQC NMR spectrum. The top and bottom panels show the same spectrum at two different signal levels. Only the major TA signals are visible in the top panel, whereas low-intensity rhamnan backbone signals are visible in the lower panel. (C) Ring region of the ^1^H/^13^C HSQC NMR spectrum. Signals A to E were found to be associated with rhamnan (as shown in Fig. 2), whereas signals F to J were found to be associated with a TA molecule linked to the rhamnan backbone.

As mentioned above, EPA was prepared in its native form (WT-Q1, Fig. 1C), composed of Rha, Glc, GlcN, GalN, and ribitol (Rbo) in an approximate ratio of 1:0.4:0.7:1.2:0.9 (Fig. S2A). Upon fractionation on a Sephadex G-50 column, WT-Q1 eluted as a single fraction that was analyzed by 1D-^1^H 2D ^1^H/^1^H and ^1^H/^13^C NMR experiments. WT-Q1 contained all anomeric signals associated with the rhamnan 2,3-αRha, t-αGlc, 3-αRha, and t-βGlcNAc residues (Fig. 3) as well as four anomeric groups of signals, absent from the rhamnan backbone spectrum (inverted triangles in Fig. 3). ^1^H/^1^H COSY, TOCSY, and ^1^H/^13^C HSQC NMR experiments allowed these signals to be further associated with five individual anomeric signals, labeled “E” to “I” and shown in Fig. 4. On the basis of their individual spin systems, “F” was identified as t-αRha, “G” as 3,6-βGalNAc, “H” as 3,4-βGalNAc, “I” as 6-βGlc, and “J” as t-αGlc. ^1^H/^13^C HSQC NMR also revealed the presence of 5-linked Rbo-1-P, in agreement with ^31^P NMR, indicating the presence of phosphodiester groups (∼1 ppm) absent from the rhamnan backbone (Fig. S3). All the ^1^H and ^13^C NMR chemical shift values identified in our study (see Table S1 in the supplemental material) were identical to those previously described for a secondary cell WTA-like polysaccharide composed of the two repeating units -6)[αLRha*p*(1-3)]βDGal*p*NAc(1-5)Rbo-1-P and βDGlc*p*(1-3)[αDGlc*p*(1-4)]βDGal*p*NAc(1-5)Rbo-1-P (14). In agreement with this finding and in contrast to WT-Q1, ^1^H NMR analysis and the monosaccharide composition of Δ*epaX*-Q1 revealed that this fraction corresponds to the rhamnan backbone previously characterized (Fig. 2; see also Fig. S2 and S4). Furthermore, as mentioned above, Δ*epaX*-Q1 treated with HF did not release any TA fragments, indicating that this fraction indeed contains only the rhamnan backbone and lacks decorations. Taken together, these results showed that EPA is composed of a rhamnan backbone decorated with TA linked by phosphodiester bonds.

10.1128/mBio.00277-20.2 FIG S2 Monosaccharide composition analysis of (A) WT-Q1 and (B) Δ*epa*X-Q1. Download FIG S2, PDF file, 0.1 MB. Copyright © 2020 Guerardel et al. 2020 Guerardel et al. This content is distributed under the terms of the Creative Commons Attribution 4.0 International license.

10.1128/mBio.00277-20.3 FIG S3 Comparison of the ^31^P spectra of polysaccharide fractions purified from the E. faecalis VE14089 WT strain. (A) Spectrum of the rhamnan backbone extracted and purified by sequential treatment with HCl and HF. (B) Spectrum of WT-Q1 purified by anion exchange chromatography following enzymatic degradation of the cell wall from E. faecalis. Download FIG S3, PDF file, 0.2 MB. Copyright © 2020 Guerardel et al. 2020 Guerardel et al. This content is distributed under the terms of the Creative Commons Attribution 4.0 International license.

10.1128/mBio.00277-20.6 TABLE S1 ^1^H and ^13^C NMR chemical shift values for teichoic acid linked to rhamnan in the WT-Q1 fraction. Download Table S1, PDF file, 0.04 MB. Copyright © 2020 Guerardel et al. 2020 Guerardel et al. This content is distributed under the terms of the Creative Commons Attribution 4.0 International license.

### EpaX is not involved in the biosynthesis of CPS.

NMR analyses of the WT-Q2 and *ΔepaX-*Q2 fractions obtained after gel filtration (Fig. 1A) showed that both fractions contained a diheteroglycan corresponding to the E. faecalis capsule described in CPS-C and CPS-D strains (11, 12). This polymer is composed of disaccharide repeating units -6)βGal-(1-3)βDGlc*p*-(1-, with O-acetylation in position 5 and lactic acid substitution at position 3 of the Gal*f* residue). As shown in Fig. S5 (see also Table S2), analysis of the spin systems led to identification of the residue as a βGalf, and the strong downfield shifts of C3 and C6 at 85.7 and 70.23 ppm, respectively, indicated substitutions in the C3 and C6 positions. Similarly, the C4 substitution of the βGlc residue was established from the downfield shifts of C6 at 83.3 ppm (Fig. S5). In line with previous work, ^1^H/^13^C HSQC analysis confirmed the presence of -CH_3_ and -CH(OH) in the lactyl group at 1.385/19.3 ppm and -CH_3_ in the acetyl group at 2.172/21.6 ppm (Fig. S5B) that were associated with -CO group of the lactyl and acetyl groups at 174.9 and 180.4 ppm, respectively, through ^3^*J*_H,C_ and ^2^*J*_H,C_ connections on the ^1^H/^13^C HMBC spectrum (Fig. S5C). The position of the acetyl group was deduced from the strong downfield shift of Galf-H5 at 5.395 ppm, whereas the sequence was deduced from NOESY and HMBC spectra (Fig. S5D). These observations indicated that EpaX had no direct role in CPS biosynthesis.

10.1128/mBio.00277-20.4 FIG S4 Comparison of the ^1^H NMR spectra of (A) WT-Q1 and (B) Δ*epa*X-Q1. Download FIG S4, PDF file, 0.1 MB. Copyright © 2020 Guerardel et al. 2020 Guerardel et al. This content is distributed under the terms of the Creative Commons Attribution 4.0 International license.

10.1128/mBio.00277-20.5 FIG S5 2D NMR analysis of CPS from E. faecalis VE14089 WT (WT-Q2) purified by anion exchange chromatography following enzymatic degradation of the cell wall. (A and B) Anomeric (A) and aliphatic regions (B) of the ^1^H/^13^C HSQC spectrum. (C) Identification of carboxyls of lactyl and acetyl groups through the ^3^*J*_H,C_ and ^2^*J*_H,C_ HMBC connection. (D) Structure of the identified CPS. Download FIG S5, PDF file, 0.1 MB. Copyright © 2020 Guerardel et al. 2020 Guerardel et al. This content is distributed under the terms of the Creative Commons Attribution 4.0 International license.

10.1128/mBio.00277-20.7 TABLE S2 ^1^H and ^13^C NMR chemical shift values for the WT-Q2 fraction corresponding to CPS. Download Table S2, PDF file, 0.05 MB. Copyright © 2020 Guerardel et al. 2020 Guerardel et al. This content is distributed under the terms of the Creative Commons Attribution 4.0 International license.

### EPA decorations are exposed at the cell surface.

**FIG 5:**
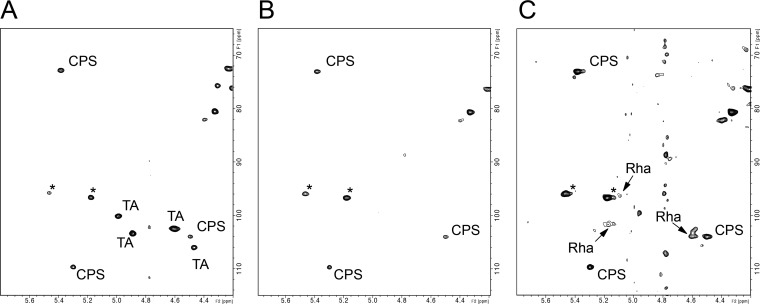
Anomeric region of the ^1^H/^13^C HSQC HR-MAS NMR spectra of intact E. faecalis bacterial cells. (A) Spectrum of the WT strain. (B and C) Spectra of the Δ*epa*X mutant. Signals were associated with the rhamnan backbone (Rha), teichoic acid decoration (TA), or the capsule polysaccharide (CPS). *, unidentified carbohydrate-associated signals. The intensities of spectra depicted in panels A and B have been adjusted according to the relative intensities of individual CPS signals to show overall similar signal levels. In contrast, panels B and C show the same spectrum at two different signal levels; only the major signals of CPS are visible in panel B, whereas the minor signals of Rha are visible in panel C.

To establish whether or not the TA decorations of the rhamnan backbone were surface exposed, we analyzed intact cells by HR-MAS NMR, a method that can be performed on intact bacterial cells (30–32). The anomeric region of the ^1^H/^13^C HSQC HR-MAS spectrum of intact WT cells showed two main groups of intense signals (Fig. 5A). These signals were identified as TA and CPS, based on comparison with analyses of purified molecules by liquid NMR analyses (Fig. 4; see also Fig. S5). Two other anomeric signals of weaker intensity were also observed but could not be identified from the complex mixture, suggesting the presence of at least one other unidentified surface polysaccharide. Surprisingly, HR-MAS spectra obtained for the WT strain did not show any signals associated with the rhamnan backbone, regardless of the signal level. The intensity of individual signals in HR-MAS NMR depends on the molecular abundance of the compounds but also on their intrinsic mobility. Our results therefore indicate that TA and CPS were flexible cell surface-exposed molecules whereas the low intensity of the rhamnan signals supports the idea that the backbone was embedded in the cell wall, reducing its mobility and surface exposure. The Δ*epaX* mutant generated a very simple spectrum containing only the signals associated with CPS and with the unidentified polysaccharide (Fig. 5B) and low-intensity rhamnan-associated signals (Fig. 5C). These observations strongly suggested that removal of TA increased the flexibility and the surface exposure of the rhamnan backbone.

## DISCUSSION

The present study established that E. faecalis EPA is a cell wall rhamnan decorated with phosphopolysaccharide chains corresponding to teichoic acids. We demonstrated that total carbohydrate preparations from the WT E. faecalis V583 contain a complex backbone composed of the repeating unit -[-2(αGlc1-3)αRha1-2αRha1-3(βGlcNAc1-2)αRha1-2αRha1-2αRha1-2αRha1-], covalently linked to the Rbo-phosphate-containing polymers previously described by Geiss-Liebisch et al. as WTA I and WTA II (14). Quantification of unsubstituted and substituted rhamnan-TA in enzymatic polysaccharide preparations from the WT showed that more than 90% of the WT rhamnan carried TA fragments. In contrast, we found that EPA in the Δ*epa*X mutant corresponds to a rhamnan backbone without any decoration, confirming our previous hypothesis that EpaX is essential for the decoration of EPA in V583 (18).

Rhamnose-containing polysaccharides have been studied extensively in streptococci, where they may functionally compensate for the lack of TA (27, 33). In streptococci, these polysaccharides consist of a linear polyrhamnose backbone of α-1,2-linked and α-1,3-linked Rha units decorated with GlcNAc in Streptococcus pyogenes and Streptococcus uberis, Glc or Gal in Streptococcus mutans, and GalNAc side chains in Streptococcus equi subsp. *zooepidermicus* (34–37). More-complex structures have been identified in Streptococcus agalactiae and Lactococcus lactis. The rhamnopolysaccharide of S. agalactiae is highly branched, with different repeating units composed of Rha, Gal, GlcNAc, and glucitol linked by phosphodiester bonds (38). Two different types of cell wall polysaccharides have been reported in L. lactis. Strain MG1363 has been found to produce a polyrhamnose made of linear trisaccharide (2-α-l-Rha-2-α-l-Rha-3-α-l-Rha) repeating units and a polysaccharide pellicle composed of repeating hexasaccharide phosphate units (31, 39, 40). The structural organization of the rhamnopolysaccharide produced by E. faecalis is different from that of the rhamnopolysaccharide produced by other Gram-positive organisms described to date since TAs are covalently linked to the EPA rhamnan backbone. Interestingly, phosphoglycerol has been detected in the recently reported glucorhamnan polysaccharide from Ruminococcus gnavus (41). This finding supports the idea that phosphodiester-linked side chain repeats on a rhamnan backbone may represent a form of structural organization existing beyond the *Lactobacillales* order (41). In addition to EPA, E. faecalis produces a capsule made of a modified disaccharide. Our HR-MAS-NMR experiment suggested that another, as-yet-unidentified polysaccharide is exposed at the cell surface. Further studies are required to investigate if this polysaccharide is the low-molecular-weight polysaccharide composed of glycerol, phosphate, and glucose reported previously by Hancock and Gilmore (28) (PS_15_ in Fig. 3). Our analyses did not identify any poly-*N*-acetylglucosamine (PNAG)-containing polymer (recently described [42]), but this may have been due to differences in growth and/or extraction protocols, notably with respect to the need to take into account the insoluble nature of PNAG at neutral pH during purification (Gerald Pier, personal communication). Given that up to 20% of the GlcNAc amino groups of the S. aureus native PNAG polymer are not *N*-acetylated, introducing positive charges that are crucial for adherence of the PNAG polymer to the bacterial surface (43, 44), it would be useful to purify and characterize the PNAG from E. faecalis cultivated under the conditions known to promote penetration of semisolid surfaces and to determine whether *epaX* is involved in PNAG biosynthesis and/or contributes to retention or binding of PNAG through the negatively charged decorations.

**FIG 6:**
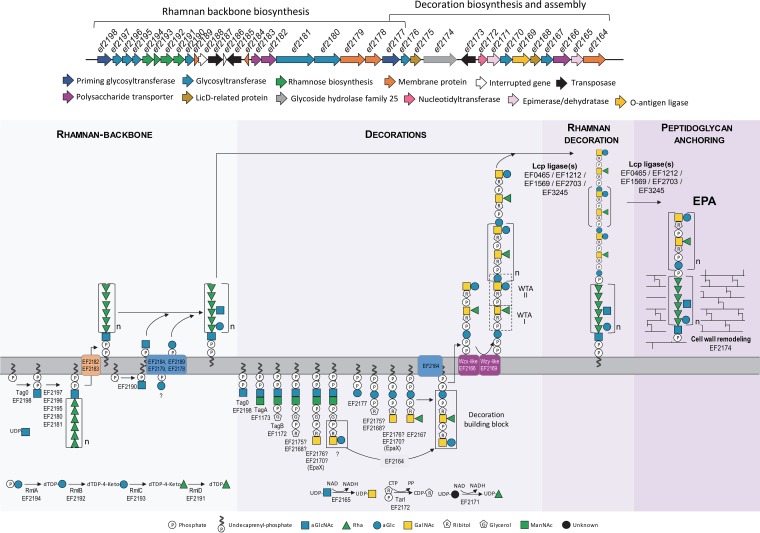
Proposed pathway for the biosynthesis of EPA in E. faecalis V583. (rhamnan backbone) Enzymes EF2194 (RmlA), EF2192 (RmlB), EF2193 (RmlC), and EF2191 (RmlD) synthesize the dTDP-l-Rha precursor. Enzyme EF2198 (TagO) initiates biosynthesis of the rhamnan by transferring GlcNAc-1-P from UDP-GlcNAc to undecaprenylphosphate. The glycosyltransferases (EF2197, EF2196, EF2195, EF2181, and EF2180) then add the subsequent Rha. ABC transporter proteins EF2182 and EF2183 transport the rhamnan across the membrane. EF2178 and EF2179 transfer Glc and GlcNAc to the rhamnan from GlcNAc-P-Und and Glc-P-P-Und, generated by EF2190 (GacI) and EF2177, respectively, with the aid of EF2184 and EF2189. (Decoration chains) Enzymes EF2165, EF2172, and EF2171 synthesize UDP-GalNAc, CDP-Rbo, and UDP-Rha, respectively. Biosynthesis of the repeat units initiates on different acceptors. The -6)[αLRha*p*(1-3)]αDGal*p*NAc(1-5)Rbo-1-P repeat unit initiates on Und-PP-Glc, whereas the βDGlc*p*(1-3)αDGal*p*NAc(1-5)Rbo-1-P repeat unit initiates on Und-PP-GlcNAc-ManNAc-GroP derived from the action of TagA (EF1173) and TagB (EF1172). The RboP is added by EF2168 or EF2175. The glycosyltransferases EF2176 and EF2170 transfer the subsequent GalNAc residues. EF2167 is likely to transfer Rha onto GalNAc. Although none of the candidate genes analyzed were predicted to be involved in the transfer of Glc to GalNAc or in the assembly of the repeat units, the units are likely to be assembled by the membrane protein EF2164 before translocation of the resulting building block across the membrane by the Wzx-like protein EF2166 and polymerization by the Wzy-like-polymerase EF2169. (Rhamnan-decoration assembly and EPA attachment) Polymerized decorations are probably ligated to the rhamnan backbone by one of LCP enzymes (EF0465, EF1212, EF1569, EF2703, and/or EF3245). The resulting EPA phosphorhamnan is transferred onto the peptidoglycan by one of the LCP enzymes aided by EF2174.

The elucidation of the complete structure of EPA allowed us to assign specific roles to the genes of the two *epa* loci encoding the rhamnan backbone (*ef2198* to *ef2177*) and its decorations (*ef2176* to *ef2164*). A hypothetical model describing the synthetic steps corresponding to the rhamnan backbone and the TA decorations and their assembly is presented in Fig. 6. The rhamnan backbone is most probably synthesized in a way similar to that previously reported for L. lactis and S. pyogenes (31, 45). Functional predictions (see Table S3 in the supplemental material) suggest that EF2194, EF2193, EF2192, and EF2191 (RmlA, C, B, and D, respectively) are responsible for the production of the l-Rha precursor dTDP-Rha (27). Following the addition of GlcNAc to the lipid carrier undecaprenyl phosphate (Und-P) by the TagO homolog EF2198 (14), we propose that EF2197, EF2196, EF2195, EF2181, and EF2180 contribute to the polymerization of the rhamnan chain on the inner face of the cytoplasm. Consistently, EF2195 is homologous to the α1,3 rhamnosyl transferase Cps2F of Streptococcus pneumoniae (46) and the two latter share homology with the carboxy-terminal domain of WsaE rhamnosyltransferase of Geobacillus stearothermophilus responsible for the formation of α1,2-Rha linkage using dTDP-l-Rha precursor (47). In the next step, we predict that the lipid-anchored rhamnan is translocated across the cytoplasmic membrane by the ABC transporter formed by EF2182 and EF2183. Once exposed at the cell surface, the rhamnan would then be modified by the transfer of Glc and GlcNAc residues as described previously for S. pyogenes (45). This would require formation of Und-P-GlcNAc and Und-P-Glc lipid intermediates by the undecaprenyl-phosphate GlcNAc transferase EF2190 and an unknown enzyme, respectively. Four proteins encoded by the *epa* conserved region with hypothetical functions (EF2178, EF2179, EF2184, and EF2189) could contribute to the transport of the Glc and GlcNAc residues bound to the lipid carrier and their transfer onto the rhamnan chain.

10.1128/mBio.00277-20.8 TABLE S3 Updated annotation of the *epa* locus genes of Enterococcus faecalis V583. Download Table S3, PDF file, 0.1 MB. Copyright © 2020 Guerardel et al. 2020 Guerardel et al. This content is distributed under the terms of the Creative Commons Attribution 4.0 International license.

Functional predictions of the *epa* decoration genes indicate the presence of a Wzx/Wzy-dependent pathway. Since both the Wzx-like flippase (*ef2166*) and the Wzy-like polymerase (*ef2169*) are encoded by a single gene, it seems most likely that a single decoration chain is transported across the cytoplasmic membrane. The model presented in Fig. 6 is based on this hypothesis and takes into account the essential roles of both EpaX (EF2170) and TagB (EF1172) in the production of TAs (14). We propose that two TA chains are synthesized on distinct lipid carriers and are linked and translocated via the Wzx flippase (Fig. 6). In our model, the synthesis of the first chain corresponding to WTA I (14) is initiated by the predicted undecaprenyl-phosphate glycosyltransferase EF2177, which is similar to initiating glycosyltransferases involved in the synthesis of Wzy-dependent polysaccharides (48). We propose that one of the putative phosphotransferases of the LicD-family, EF2175 or EF2168, is transferring Rbo-phosphate on the glucose-undecaprenyl carrier using CDP-Rbo synthesized by the TarI homolog EF2172 as a substrate (49). This step is followed by the addition of a GlcNAc residue by either EF2176 or EF2170 (EpaX) and of a Rha residue by the putative rhamnosyltransferase EF2167. The substrates required for these two synthetic steps (UDP-GlcNAc and UDP-Rha) are likely to be produced by the UDP-*N*-acetylglucosamine 4-epimerase EF2165 (50) and the epimerase/dehydratase EF2171, respectively. According to our model, the synthesis of the second chain (14) is initiated by the undecaprenyl-phosphate glucose phosphotransferase EF2177 and involves TagA (EF1173), which adds a mannosyl residue to the Und-PP-GlcNAc precursor. The glycerophosphotransferase TagB then transfers a glycerol-phosphate unit to generate an Und-PP-GlcNAc-ManNAc-GroP intermediate. We propose that this molecule is used as an acceptor for the addition of a ribitol-phosphate residue by either of the LicD homologs EF2175 and EF2168, followed by the addition of GalNAc by either EF2176 or EF2170 (EpaX). The transfer of a Glc residue by an as-yet-unidentified transferase completes the synthesis of the first part of the decoration molecule. We hypothesize that, once the two TA chains have been synthesized, they are linked by the membrane protein EF2164, thereby generating a complete decoration building block. This molecule is then translocated to the outer face of the cytoplasmic membrane by the Wzx flippase (EF2166) and polymerized by the Wzy-like-polymerase EF2169. The latter has 11 transmembrane helices, a structure reminiscent of the predicted Cap5J polymerase from Staphylococcus aureus (51) and of the WaaL O antigen ligases that transfer O antigen polysaccharides onto the lipid A core in Gram-negative bacteria (52). Polymerization generates the previously identified WTA II (14). The addition of the polymerized TA molecule on the rhamnan chain is expected to be performed by one of the five LCP (LytR-CpsA-Psr) protein candidates (EF0465, EF1212, EF1569, EF2703, and EF3245) encoded outside the *epa* locus (53, 54). Following the covalent attachment of the decoration chains on the rhamnan backbone, the complete EPA polymer is anchored on the peptidoglycan molecule via one of the LCP enzymes cited above. The remaining gene, which remains to be included in our synthetic model, encodes EF2174, a predicted peptidoglycan hydrolase belonging to glycosyl hydrolase family 25. It is likely to play a role in cell wall remodeling to facilitate the assembly of EPA in the cell wall. The independent syntheses of the core rhamnan backbone and the decoration chains and their assembly at the outer face of the cytoplasmic membrane are reminiscent of the biosynthesis of lipopolysaccharides in Gram-negative bacteria (55). However, the individual biosynthetic steps proposed in Fig. 6 remain to be experimentally tested. It would also be interesting to determine whether the decoration chains are exclusively bound to rhamnan or are also anchored to peptidoglycan.

The idea that the rhamnose-containing cell wall polysaccharides obtained by acid hydrolysis correspond to a group-specific polysaccharide antigen for group D streptococci, a group to which enterococci belongs, was first proposed by S. D. Elliot in 1959 (66). Elliot also hypothesized that these cell wall polysaccharides were deeply buried in the cell wall. Our study strongly supports the idea that EPA decorations are exposed at the surface of bacterial cells whereas the rhamnan backbone is embedded within the cell wall, as previously reported for L. lactis rhamnan (31). Given the genetic diversity of the loci encoding the enzymes involved in EPA decoration, it is tempting to propose that these decorations correspond to the group D type-specific polysaccharide antigen. A similar *epa* locus was identified previously in Enterococcus faecium (26), suggesting that a similar form of structural organization may be present in this species also. Even if the reported decorations are specific to V583-related strains, this work provides a conceptual framework to study the EPA structural and functional relationships in enterococci. Most *epa* variable regions do not encode a gene that can be clearly identified as an *epaX* ortholog. In addition, a variable number of glycosyltransferases are present in the decoration locus. The precise roles of EpaX and homologs remain to be investigated. Elucidation of the complete EPA structure has direct implications for our understanding of the organization of the enterococcal cell surface and how this organization influences the interaction with the host. Both CPS and EPA play a role in immune evasion. CPS confers resistance to complement-mediated opsonophagocytosis, likely by masking bound C3b (10). EPA has been shown to protect unencapsulated strains from opsonophagocytic killing by polymorphonuclear leukocytes and from phagocytosis in zebrafish (21, 24, 56). More recently, Geiss-Liebisch et al. showed that the decoration chains of strain V583, formerly considered to represent TA, protect against complement-mediated phagocytic killing by conferring resistance to the binding of the mannose that binds lectin and thus interfering with complement deposition by the lectin pathway (14). The finding that EPA decorations are cell surface exposed is in line with the critical role of these decorations in protecting E. faecalis against phagocytosis and with their role in the interaction of the bacteria with the biotic and abiotic environment. The diversity of the *epa* decoration loci suggests that changes in decoration structure, and eventually in their charge, are likely to influence fitness and survival. This report is the first to provide the complete structure of the EPA rhamnan backbone and to identify the link between teichoic acids and EPA decoration, clarifying the picture of enterococcal cell wall polysaccharide organization. Future genetic and biochemical studies will help to increase our understanding of the effect of gene variability on biochemical and structural diversity and of how this diversity affects the role of EPA.

## MATERIALS AND METHODS

### Bacterial strains and growth conditions.

E. faecalis VE14089 is a plasmid-cured derivative of V583 (57); the Δ*epaX* mutant is isogenic to VE14089 and has a deletion in *epaX* (18). E. faecalis strains were grown on GM17 agar plates or in M17 broth (Difco) supplemented with 0.5% glucose (GM17) at 37°C without aeration.

### Polysaccharide extraction and purification.

Enzymatic preparations of cell wall polysaccharide were performed essentially as described previously (18). Cells were grown in 1-liter cultures in GM17 and harvested 1 h after the stationary phase. They were then washed in phosphate-buffered saline (PBS) and treated with lysozyme and mutanolysin and then with DNase, RNase, and proteinase K as described in reference 18. Proteins were extracted with phenol-chloroform-isoamyl alcohol (25:24:1) and chloroform. The crude preparation was then dialyzed and lyophilized. Samples (10 mg) of each preparation were fractionated on a Q Sepharose Fast Flow anion exchange column (Cl form, 1 cm by 10 cm), equilibrated in water. Compounds were eluted with water followed by a linear gradient of NaCl (0 to 1 M, 60 ml). Fractions were then assayed for total sugars (58). Carbohydrate-positive fractions were pooled, concentrated, and desalted on a Sephadex G-50 column. Gel filtration was carried out on a Sephadex G-50 column (1 cm by 40 cm and 2.6 cm by 90 cm) and a Bio-Gel P2 column (2.6 cm by 60 cm). Compounds were eluted with 0.01% acetic acid (AcOH). Aliquots of each fraction were assayed for neutral sugars (58).

The rhamnan backbone was prepared essentially as described previously (59). Cells were treated with 5% trichloroacetic acid (TCA) for 48 h at 5°C with stirring. After centrifugation, the supernatant was dialyzed and freeze-dried to give the TCA extract. Cell debris was extracted by consecutive treatments with 0.01 N HCl at 100°C for 20 min and 0.1 N HCl at 100°C for 20 min. These HCl extracts were then pooled and deproteinated by addition of TCA and then dialyzed and freeze-dried. The rhamnan backbone was prepared for NMR analysis by treating 20 mg of the 0.01 M HCl extract with 100 μl of 48% HF at 4°C for 24 h. After evaporation of the HF, the rhamnan backbone was purified by gel filtration chromatography on a Sephadex G-50 column.

### Analytical methods.

Monosaccharides were identified and quantified as reduced and acetylated derivatives (alditol acetates) relative to internal *myo*-inositol standards. Polysaccharide samples (0.2 to 1 mg) were hydrolyzed with 4 M trifluoroacetic acid (TFA) at 110°C for 3 h and then dried. They were then placed in water, and a few drops of 0.1 M NH_4_OH (pH 9) were added before the samples were reduced with NaBH_4_. Any excess NaBH_4_ was removed by addition of 10% AcOH–methanol (MeOH), and the solution was dried under a stream of nitrogen. The drying process was repeated twice more after addition of 10% AcOH–MeOH (1 ml) and a further two times after addition of MeOH (1 ml). The residue was acetylated with 0.4 ml of acetic anhydride and 0.4 ml of pyridine at 100°C for 1 h and was then dried under a stream of nitrogen with addition of toluene (1 ml) before being analyzed by GC-MS.

The absolute configurations of the monosaccharides were determined by GC-MS analysis of acetylated 2-octyl glycoside derivatives, as described previously (59).

Methylation was performed using the Ciucanu-Kerek procedure (60) modified by Read et al. (61). The polysaccharide (0.5 to 1 mg) was dissolved in 1 ml of dimethyl sulfoxide. Freshly powdered NaOH (about 50 mg) was added, and the mixture was stirred for 15 min. Methyl iodide (0.2 ml) was then added and the mixture stirred for another 2 h. The reaction was stopped by addition of 3 ml of 10% aqueous Na_2_S_2_O_3_. The permethylated product was extracted twice with CHCl_3_ (2 ml). The organic phase was washed five times with water (4 ml), filtered through a cotton-plugged Pasteur pipette, and evaporated. The product was hydrolyzed with 4 M TFA (110°C, 3 h), dried, reduced with NaBD_4_, and then converted into alditol acetates before being analyzed by GC-MS as described above. Methylated derivatives were identified using the Complex Carbohydrate Research Center partially methylated alditol acetates database (www.ccrc.uga.edu/specdb/ms/pmaa/pframe.html) and by comparison with the authentic standards of methylation analysis of polysaccharides of various L. lactis strains.

Gas chromatography (GC) was performed on a Trace GC Ultra system (Thermo Scientific) equipped with a NMTR-5MS capillary column (30 m by 0.25 mm) and a flame ionization detection (FID) unit using a temperature gradient of 170°C (3 min) → 250°C at 5°C min^−1^. GC-MS was performed using a Trace GC Ultra system TSQ quantum GC detector (Thermo Scientific), equipped with a SILGEL1MS capillary column (30 m by 0.25 mm), and a temperature gradient of 170°C → 230°C at 3°C min^−1^ → 270°C at 10°C min^−1^ (10 min).

### Nuclear magnetic resonance studies.

Samples were solubilized in highly enriched deuterated water (99.96% deuterium; EurisoTop, St-Aubin, France) and lyophilized. This process was repeated twice. Data were recorded on a 14.1-T spectrometer (Institut Pasteur de Lille) and a 21.4-T spectrometer (Unité de Glycobiologie Structurale et Fonctionnelle, Infrastruture de Recherche-Très Hauts Champs-Résonance Magnétique Nucléaire, CNRS); protons resonated at 600 and 900 MHz, and ^13^C resonated at 151 and 250 MHz, respectively. The 14.1-T spectrometer was equipped with a 5-mm-diameter quadruple-resonance inverse (QCI) cryoprobe head with ^1^H, ^2^H, ^19^F, and ^13^C cooled channels and a ^15^N channel with a z-gradient. The 21.4-T spectrometer was equipped with a 5-mm-diameter triple-resonance inverse (TCI) cryoprobe with ^1^H, ^2^H, and ^13^C cooled channels and a ^15^N channel with a z-gradient. All samples were put in 5-mm tubes with matching amounts of D_2_O. Acetone was added as an internal standard, starting from a solution of 2.5 μl of acetone–10 ml of D_2_O. All pulse sequences were taken from the Bruker library of pulse programs and then optimized for each sample. Spectral widths were 12 and 200 ppm for the proton and carbon observations, respectively. TOCSY was performed with various mixing times of 40 to 120 ms, and N spectra were recorded with a mixing time of 300 ms. Edited ^1^H-^13^C HSQC spectra were recorded with 1,536 data points for detection and 256 data points for indirect direction.

HR-MAS NMR experiments were conducted using an 18.8-T Avance Neo Bruker spectrometer. Data were acquired with a ^1^H/^13^C/^31^P/^2^H probe with uniaxial gradients. Before analysis, cell pellets were washed twice with deuterium oxide (Euriso-top, Gif-sur-Yvette, France). Cell pellets (50 μl), together with 0.5 μl of acetone as an internal standard, were centrifuged at 3,000 rpm in 4-mm ZrO_2_ rotors (CortecNet, Paris, France) (29). All spectra were recorded at 300 K, and the rotor spinning rate was 8 kHz. All pulse programs were sourced from the Bruker library, and delays and powers were optimized for each sample. For ^1^H-^13^C HSQC experiments, the spectral widths were 12,820 Hz (^1^H) with 1,024 points for the FID resolution and 29,994 Hz (^13^C) during 400 scans, giving 12.5 Hz/pt and 75.0 Hz/pt, respectively.

### Protein sequence analysis.

Protein sequence homology was analyzed using NCBI BLASTp (62). Protein domains were identified by searching the Pfam and SMART databases (63, 64). Putative transmembrane helices were predicted using the TMHMM2.0 program (65).
