# Supplementary figures and images for: Complete Structure of the Enterococcal Polysaccharide Antigen (EPA) of Vancomycin-Resistant Enterococcus faecalis V583 Reveals that EPA Decorations Are Teichoic Acids Covalently Linked to a Rhamnopolysaccharide Backbone
Source: mBio. 2020 Apr 28;11(2):e00277-20. doi: 10.1128/mBio.00277-20 (PMC7188991; doi:10.1128/mBio.00277-20)

**Figure S1**

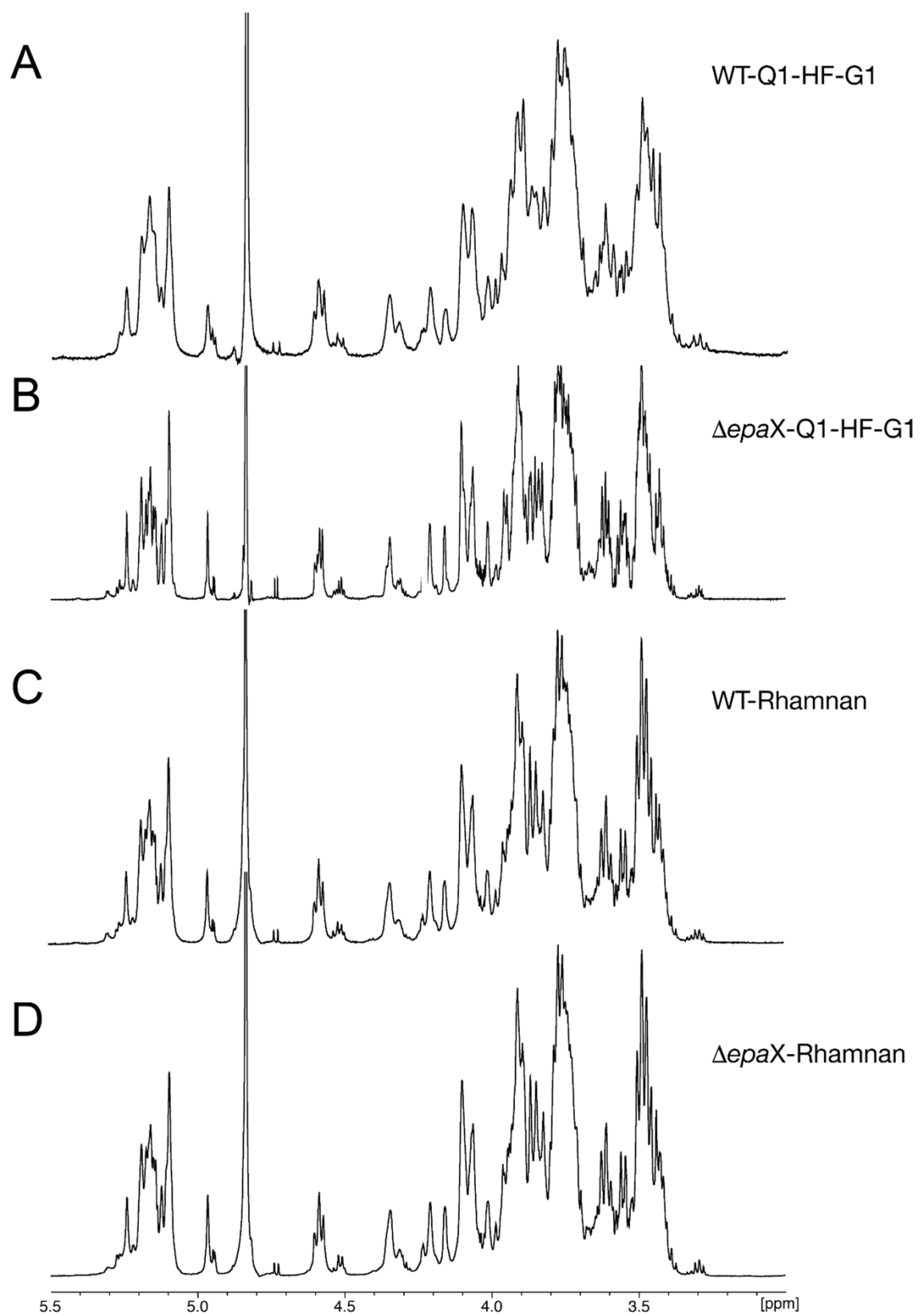

Supplement: FIG S1 [file mBio.00277-20-sf001.pdf]

Figure S2

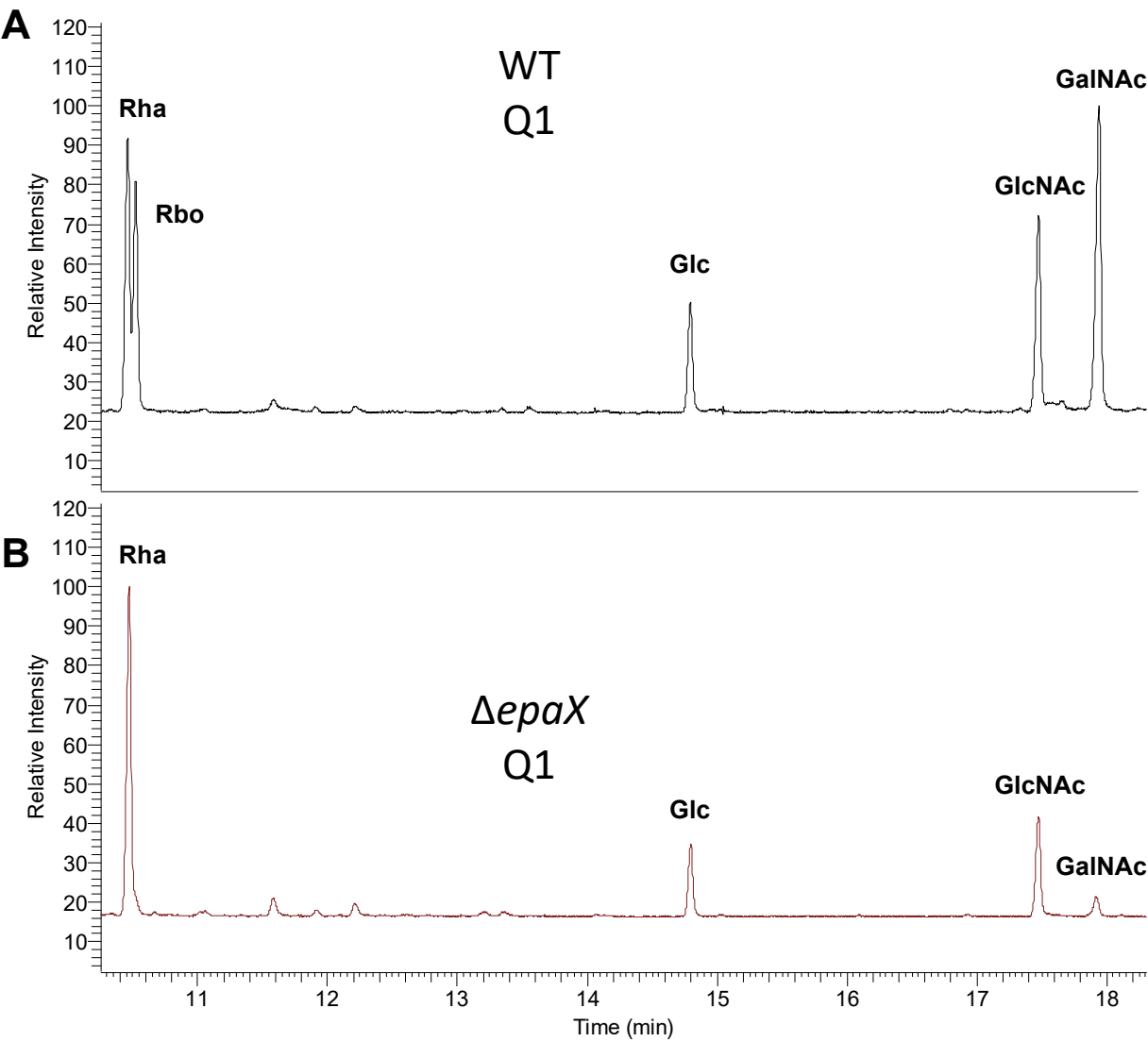

Supplement: FIG S2 [file mBio.00277-20-sf002.pdf]

Figure S3

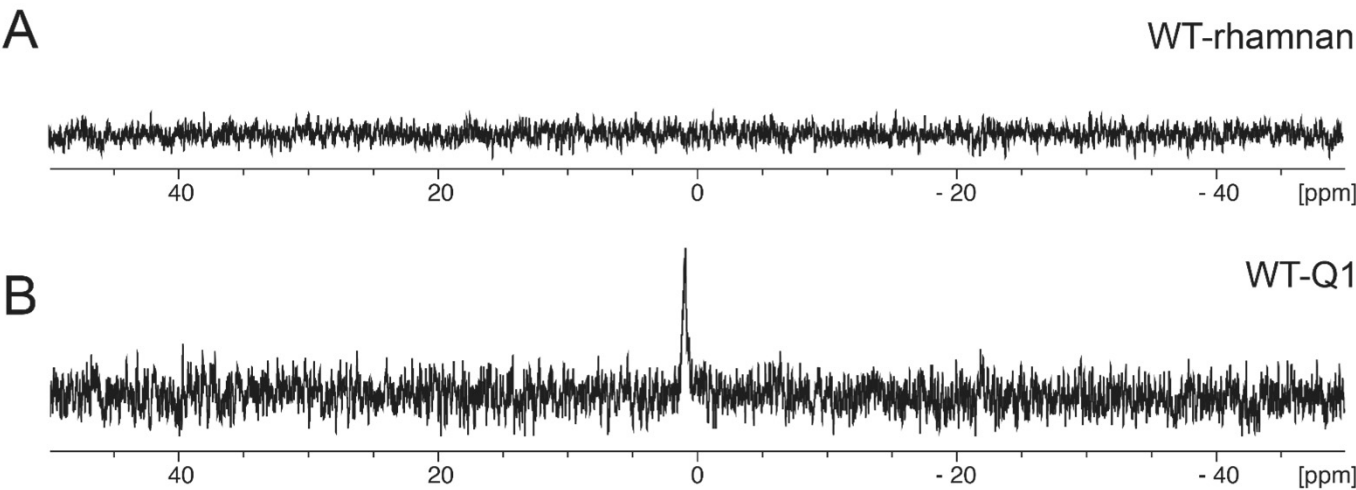

Supplement: FIG S3 [file mBio.00277-20-sf003.pdf]

**Figure S4**

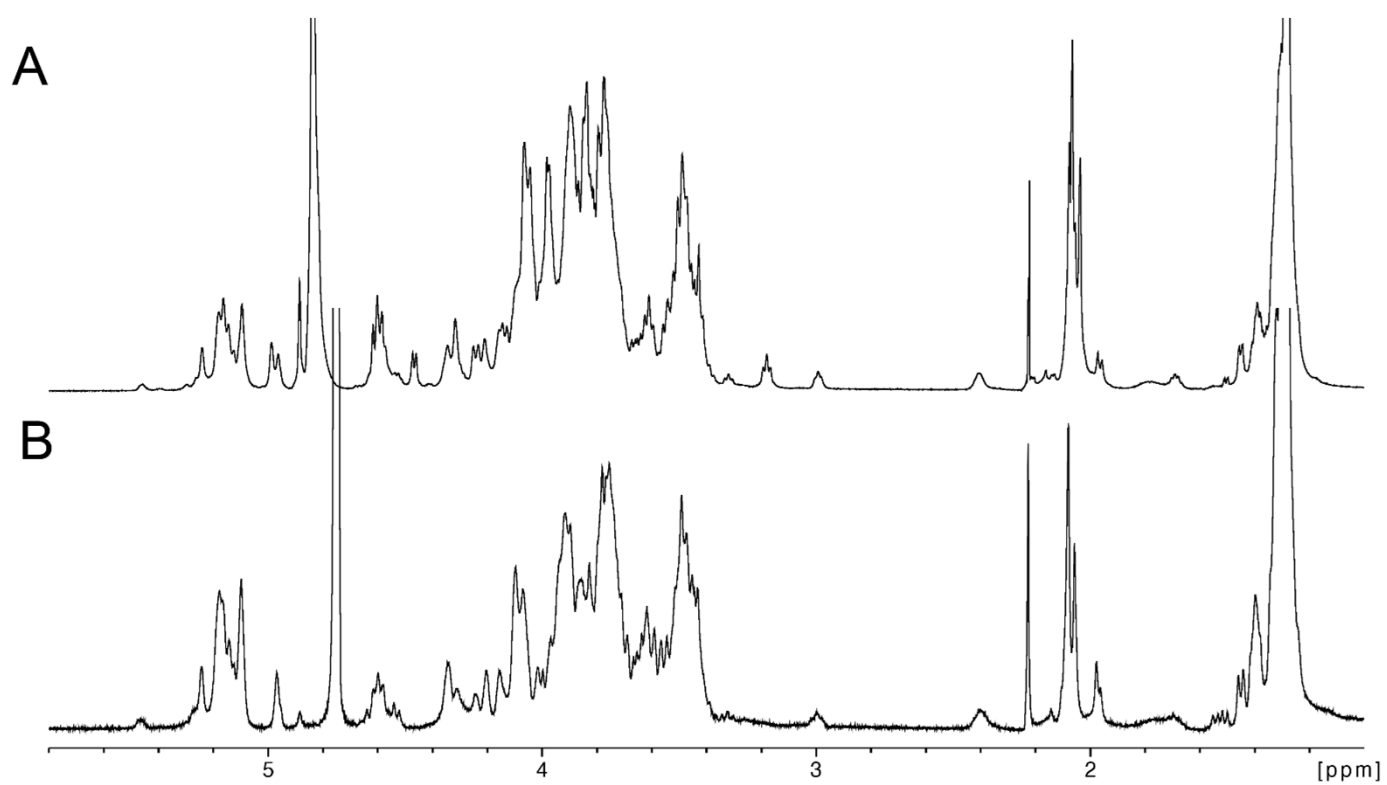

Supplement: FIG S4 [file mBio.00277-20-sf004.pdf]

**Figure S5**

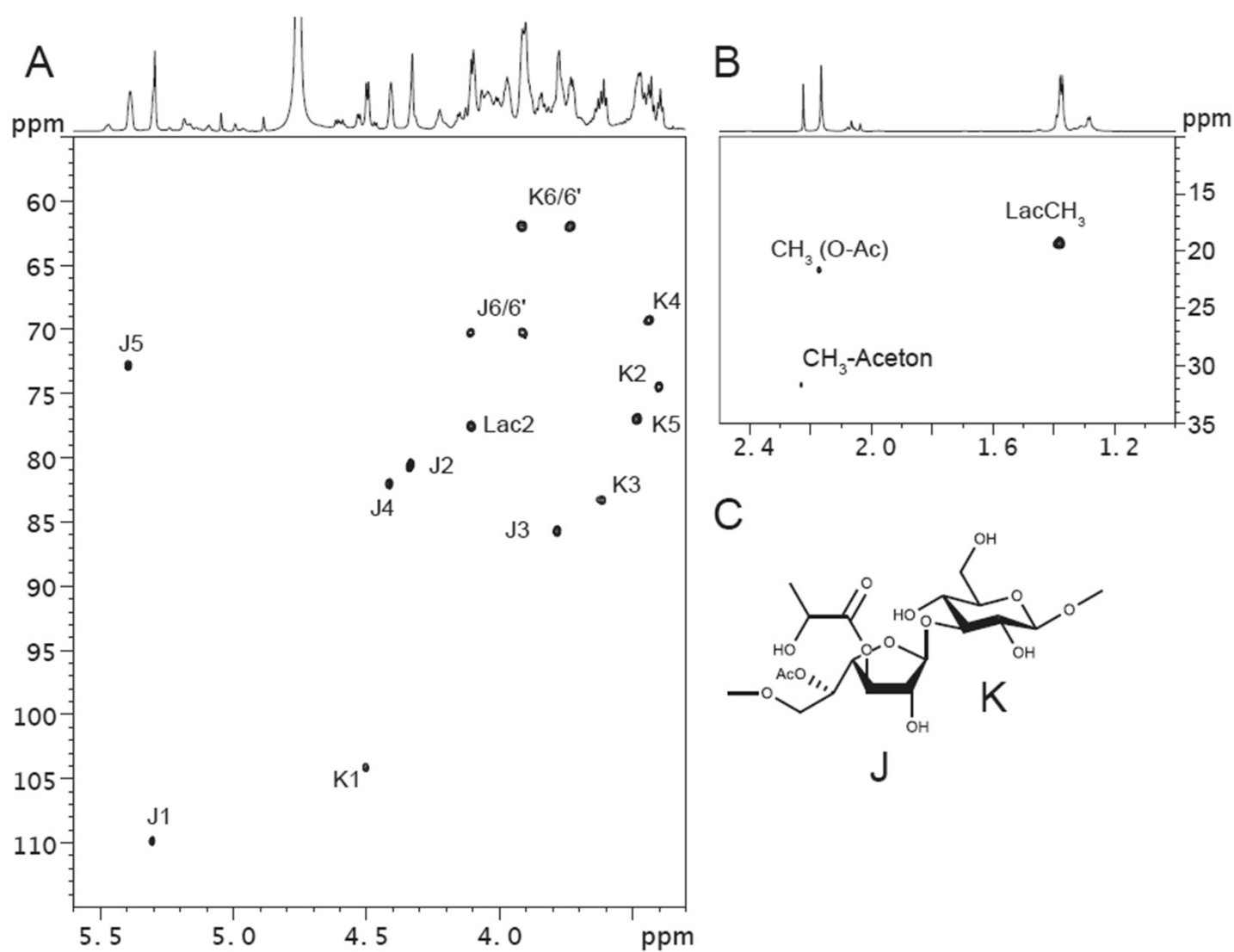

Supplement: FIG S5 [file mBio.00277-20-sf005.pdf]
