# Supplementary material for: Complete Structure of the Enterococcal Polysaccharide Antigen (EPA) of Vancomycin-Resistant Enterococcus faecalis V583 Reveals that EPA Decorations Are Teichoic Acids Covalently Linked to a Rhamnopolysaccharide Backbone
Source: mBio. 2020 Apr 28;11(2):e00277-20. doi: 10.1128/mBio.00277-20 (PMC7188991; doi:10.1128/mBio.00277-20)
Supplement: TABLE S1 [file mBio.00277-20-st001.pdf]

Table S1: ^{1}H and ^{13}C NMR chemical shift values of teichoic acid linked to rhamnan in WTQ1 fraction.

Residue	Chemical shifts ^{1}H and ^{13}C [δ]						
	H1<br>C1	H2<br>C2	H3<br>C3	H4<br>C4	H5<br>C5	H6^{a}<br>C6	H6^{b}<br>C6
t-α-Rha*p*-<br>F	4.887<br>103.66	3.840<br>71.39	3.804<br>71.19	3.432<br>73.13	3.825<br>70.39	1.284<br>17.85	
→3,6)-β-GalNAc*p*-<br>G	4.591<br>102.83	4.044<br>52.76	3.789<br>79.99	4.072<br>68.43	3.877<br>74.63	4.046<br>64.97	4.046<br>64.97
→4,6)-β-GalNAc*p*-<br>H	4.611<br>102.73	4.127<br>53.09	3.913<br>80.18	4.317<br>75.68	3.796<br>76.66	3.886<br>61.18	3.828<br>61.18
→6)-β-Glc*p*-<br>I	4.468<br>106.32	3.182<br>74.18	3.476<br>76.63	3.518<br>70.30	3.520<br>75.66	4.155<br>65.62	4.074<br>65.62
t-α-Glc*p*-<br>J	4.992<br>100.29	3.497<br>73.15	3.850<br>73.79	3.507<br>7.34	4.232<br>72.45	3.895<br>61.28	3.833<br>61.28
→5)-Ribol	3.983/4.053<br>67.79	3.766<br>72.61	3.903<br>71.82	3.969<br>71.76	3.997/3856<br>71.89		
