# Supplementary material for: Complete Structure of the Enterococcal Polysaccharide Antigen (EPA) of Vancomycin-Resistant Enterococcus faecalis V583 Reveals that EPA Decorations Are Teichoic Acids Covalently Linked to a Rhamnopolysaccharide Backbone
Source: mBio. 2020 Apr 28;11(2):e00277-20. doi: 10.1128/mBio.00277-20 (PMC7188991; doi:10.1128/mBio.00277-20)
Supplement: TABLE S2 [file mBio.00277-20-st002.pdf]

Table S2: ^{1}H and ^{13}C NMR chemical shift values of WT-Q2 fraction that corresponds to CPS.

**Residue**	**Chemical shifts ^{1}H and ^{13}C [δ]**						
	H1<br>C1	H2<br>C2	H3<br>C3	H4<br>C4	H5<br>C5	H6[a]<br>C6	H6[b]
→6)-β-Gal*f*-<br>A	5.297<br>109.74	4.330<br>80.52	3.778<br>85.67	4.408<br>81.94	5.391<br>72.78	3.908<br>70.21	4.103<br>70.21
→3)-β-D-Glc*p*-<br>B	4.498<br>104.02	3.398<br>74.44	3.612<br>83.21	3.433<br>69.24	3.479<br>76.90	3.731<br>61.94	3.914<br>61.94
Lactic acid<br>LA	180.33	4.101<br>77.49	1.381<br>19.23				
O-Ac	174.85	2.167<br>21.49					
