# Supplementary material for: Complete Structure of the Enterococcal Polysaccharide Antigen (EPA) of Vancomycin-Resistant Enterococcus faecalis V583 Reveals that EPA Decorations Are Teichoic Acids Covalently Linked to a Rhamnopolysaccharide Backbone
Source: mBio. 2020 Apr 28;11(2):e00277-20. doi: 10.1128/mBio.00277-20 (PMC7188991; doi:10.1128/mBio.00277-20)
Supplement: TABLE S3 [file mBio.00277-20-st003.pdf]

Table S3: Updated annotation of *epa* locus genes of *Enterococcus faecalis* V583

Protein	Size (AA)	Annotated prediction (UniProt); Predicted domains(s)[a]	Functionaly characterized homologs[b,c]	Predicted function	Blast prot against ref prot exclusion *Enterococcaceae*
EF2198 (EpaA)	378	Glycosyl transferase, group 4 family protein; 11 TMH, PF00953	**UDP-GlcNAc:undecaprenyl phosphate N-acetylglucosaminyl 1-P transferase TagO** (358AA) of *Bacillus subtilis* subsp. *subtilis* str. 168: 40% (145/361) amino acid identity and (64%) (233/361) amino acid similarit (Soldo et al, 2002)	Transfers GlcNAc-P onto undecaprenyl-phosphate	Undecaprenyl/decaprenyl-phosphate alpha-N-acetylglucosaminyl 1-phosphate transferase [*Carnobacterium divergens*] Sequence ID: WP_051915676.1 (366 AA) 222/365(61%) 287/365(78%) 2/365(0%)
EF2197 (EpaB)	262	Glycosyl transferase, group 2 family protein; PF10111	**α 1-3 rhamnosyl transferase WsaD** (289 AA) of *Geobacillus stearothermophilus NRS 2004/3a:* 27% (66/244) amino acid identity and 48% (119/244) amino acid similarity (Steiner et al, 2008).	Transfers the first rhamnose to the lipid linked und-PP-Glc intermediate	Glycosyltransferase family 2 protein [*Carnobacterium divergens*] Sequence ID: WP_135022433.1 (260 AA) 153/260(59%) 194/260(74%) 0/260(0%)
EF2196 (EpaC)	275	Glycosyl transferase, group 2 family protein; PF00535	**Predicted rhamnosyltransferase LGG_00279** (273 AA, RfbF) of *Lactobacillus rhamnosus* GG 35% (88/254) amino acid identity and 51% (132/254) amino acid similarity (Sanchez-Rodriguez et al, 2014).	Transfers rhamnose	Glycosyltransferase [*Bacillus thermoamylovorans*] Sequence ID: WP_041848106.1 (282 AA) 115/281(41%) 167/281(59%) 9/281(3%)
EF2195 (EpaD)	237	Glycosyl transferase, group 2 family protein; PF00535	**α 1-3 rhamnosyltransferase Cps2F** (305 AA) of *Streptococcus pneumoniae* D39: 35% (75/215) amino acid identity and 53% (116/215) amino acid similarity (James et al, 2013).	Catalyzes the formation of α 1,3-Rha linkages	Glycosyltransferase family 2 protein [*Bacillus niacini*] Sequence ID: WP_034674026.1 (238 AA) 111/221(50%) 152/221(68%) 0/221(0%)
EF2194 (EpaE, RfbA)	288	Glucose-1-phosphate thymidylyltransferase; PF12804		Converts Glc-1-phosphate into dTDP-Glc	Glucose-1-phosphate thymidylyltransferase RfbA [*Carnobacterium divergens*] Sequence ID: WP_074401346.1 (290 AA) 254/286(89%) 271/286(94%) 0/286(0%)
EF2193 (EpaF, RfbC)	190	dTDP-4-dehydrorhamnose 3,5-epimerase; PF00908		Converts dTDP-4-keto-6-deoxy-D-Glc into dTDP-4-keto-L-Rha	dTDP-4-dehydrorhamnose 3,5-epimerase [*Lactobacillus mali*] Sequence ID: WP_056990349.1 (193 AA) 153/190(81%) 172/190(90%) 0/190(0%)
EF2192 (EpaG, RfbB)	342	dTDP-glucose 4,6-dehydratase; PF16363		Oxidizes and dehydrates dTDP-Glc into dTDP-4-keto-6-deoxy-D-Glc	dTDP-glucose 4,6-dehydratase [*Carnobacterium alterfunditum*] Sequence ID: WP_034545369.1 (342 AA) 294/342(86%) 317/342(92%) 0/342(0%)
EF2191 (EpaH, RmlD)	299	dTDP-4-dehydrorhamnose reductase; PF04321		Reduces dTDP-4-keto-L-Rha into dTDP-6-Rha	dTDP-4-dehydrorhamnose reductase [*Carnobacterium maltaromaticum*] Sequence ID: WP_010051523.1 (280 AA) 182/279(65%) 233/279(83%) 1/279(0%)
EF2190 (EpaI)	241	Glycosyl transferase, group 2 family protein; PF00535	**UDP-GlcNAc:Und-P GlcNAc transferase GacI** or M5005_Spy0610 (239 AA) of *Streptococcus pyogenes* 46% (113/247) amino acid identity and 59% (148/247) amino acid similarity (Rush et al 2017)	Transfers GlcNAc from UDP-GlcNAc to Und-P to yield GlcNac-P-Und.	Glycosyltransferase family 2 protein [*Streptococcus suis*] Sequence ID: WP_024413034.1 (238 AA) 157/241(65%) 193/241(80%) 3/241(1%)
EF2189 (EpaJ)	121	Uncharacterized protein; 3 TMH, DUF2304 (PF10066)		Aids EF2190	DUF2304 domain-containing protein [*Streptococcus suis*] Sequence ID: WP_044760012.1 (118 AA) 50/117(43%) 79/117(67%) 1/117(0%)
EF2188 interupted by an IS256	234	Racemase domain protein; 7 TMH, PF01757		Not assigned because of the IS insertion	Acyltransferase family protein [*Lactobacillus plantarum*] Sequence ID: WP_080373076.1 (353 AA) 63/175(36%) 96/175(54%) 9/175(5%)
EF2184 (EpaK)	113	Uncharacterized protein; 4 TMH		Aids for transfer of Glc on the polyrhamnose	EamA family transporter [*Eubacterium limosum*] Sequence ID: WP_058693542.1 (117 AA) 48/113(42%) 79/113(69%) 0/113(0%)
EF2183 (EpaL)	264	Transport permease protein; 6 TMH, PF01061		Transports the polyrhamnose to the external face of the cytoplasmic membrane	ABC transporter permease [*Eubacterium limosum*] Sequence ID: WP_058693533.1 (268 AA) 164/268(61%) 216/268(80%) 4/268(1%)
EF2182 (EpaM)	405	ABC transporter, ATP-binding protein; PF14524, PF00005		Catalyses transport of polyrhamnose	ABC transporter ATP-binding protein [*Eubacterium* sp. AM05-23] Sequence ID: WP_118518606.1 (401 AA) 289/399(72%) 345/399(86%) 0/399(0%)
EF2181 (EpaN)	1047	Glycosyl transferase, group 2 family protein; IPR029063, PF00535	**α 1-2, α 1-3 bifunctional rhamnosyl transferase WsaE** (1127 AA) of *Geobacillus stearothermophilus NRS 2004/3a:* 40% (198/500) of amino acid identity and 57% (288/500) of amino acid similarity (Steiner et al, 2008).	Catalyzes the formation of α 1,2-Rha linkages	Glycosyltransferase [*Eubacterium callanderi*] Sequence ID: WP_073383644.1 (1092 AA) 453/1092(41%) 654/1092(59%) 55/1092(5%)
EF2180 (EpaO)	713	Glycosyl transferase, group 2 family protein; 2 PF00535 domains	**α 1-2, α 1-3 bifunctional rhamnosyl transferase WsaE** (1127 AA) of *Geobacillus stearothermophilus NRS 2004/3a:* 47% (276/592) of amino acid identity and 62% (370/592) of amino acid similarity (Steiner et al, 2008).	Catalyzes the formation of α 1,2-Rha linkages	Glycosyltransferase family 2 protein [*Listeria monocytogenes*] Sequence ID: WP_075491555.1 (712 AA) 408/713(57%) 514/713(72%) 6/713(0%)
EF2179 EpaP)	656	Uncharacterized protein; 13 TMH		Participates to transfer Glc or GlcNAc on polyrhamnose	Hypothetical protein [*Lactobacillus plantarum*] Sequence ID: WP_063485480.1 (648 AA) 262/644(41%) 399/644(61%) 10/644(1%)
EF2178 (EpaQ)	433	Membrane protein, putative; 12 TMH		Participates to transfer Glc or GlcNAc on polyrhamnose	Hypothetical protein [*Paenibacillus* sp. ASL46] Sequence ID: WP_138494814.1 (401 AA) 83/297(28%) 140/297(47%) 26/297(8%)
EF2177 (EpaR)	465	Bacterial sugar transferase; 5 TMH, PF02397	**Undecaprenyl-phosphate glucose phosphotransferase WsaP** (471AA) of *Geobacillus stearothermophilus* 35% (134/388) amino acid identity and 54% (211/388) amino acid similarity (Steiner et al, 2007).	Transfers Glc from UDP-Glc to Und-P-P to yield Glc-P-P-Und	Sugar transferase [*Lactobacillus manihotivorans*] Sequence ID: WP_056964297.1 (466 AA) 270/447(60%) 360/447(80%) 0/447(0%)
EF2176 (EpaS)	252	Glycosyl transferase, group 2 family protein; PF00535	**UDP-Glc:alpha-D-GlcNAc-diphosphoundecaprenol beta-1,3-glucosyltransferase WfgD** of *Escherichia coli* B5L3F2 41% (104/253) amino acid identity and 56% (142/253) amino acid similarity (Brockhausen e al, 2008).	Transfers GalNac on a TA chain	Glycosyltransferase family 2 protein [*Arcobacter ebronensis*] Sequence ID: WP_129086999.1 (248 AA) 113/247(46%) 166/247(67%) 1/247(0%); glycosyltransferase family 2 protein [Carnobacterium divergens] Sequence ID: WP_135020075.1 (249 AA) 116/242(48%) 161/242(66%) 4/242(1%)
EF2175 (EpaT)	282	LicD-related protein; PF04991		Adds Rbo-P on a TA chain	2-C-methyl-D-erythritol 4-phosphate cytidylyltransferase [*Parvimonas micra*] Sequence ID: WP_118058863.1 (281 AA) 161/274(59%) 197/274(71%) 0/274(0%)
EF2174 (EpaU)	893	Uncharacterized protein; Signal peptide, Glycoside hydrolase family 25 (PF01183)		Participates to peptidoglycan remodelling	Hypothetical protein [Weissella bombi] Sequence ID: WP_092461148.1 (678AA) 116/318(36%) 171/318(53%) 16/318(5%) on the first 363 amino acid residues of EF2174; Hypothetical protein [*Brochothrix thermosphacta*] WP_119946283.1 (724 AA) 212/536(40%) 306/536(57%) 20/536(3%) on the last ~500 amino acids of EF2174
EF2172 (EpaV, ispD)	234	2-C-methyl-D-erythritol 4-phosphate cytidylyltransferase; PF01128	**Ribitol-5-phosphate cytidylyltransferase TarI** (235 AA) of *Streptococcus pneumoniae*: 30% (74/241) amino acid similarity and 47% (115/241) amino acid similarity (Baur et al 2009).	Transfers the cytidylyl group of CTP to D-ribitol 5-phosphate to yield CDP-ribito	2-C-methyl-D-erythritol 4-phosphate cytidylyltransferase [*Lactobacillus porcinae*] Sequence ID: WP_125709417.1 (234AA) 173/234(74%) 205/234(87%) 0/234(0%)
EF2171 (EpaW)	352	Epimerase/dehydratase, putative; PF01370		Converts an unknown substrate to UDP-Rha	NAD-dependent epimerase/dehydratase family protein [*Lactobacillus paracasei*] Sequence ID: WP_016366206.1 (353 AA) 203/352(58%) 264/352(75%) 1/352(0%)
EF2170 (EpaX)	324	Glycosyl transferase, group 2 family protein; PF00535	**UDP-Glc:GalNAcα-diphosphatelipid β1,3-Glc-transferase** WbdN (260 AA) of *Escherichia coli* O157: 30% (60/202) amino acid identity and 53% (108/202) amino acid similarity (Gao et al, 2012)	Transfers GalNac on a TA chain	Glycosyltransferase [*Lactobacillus camelliae*] Sequence ID: WP_056989625.1 (319 AA) 196/318(62%) 248/318(77%) 2/318(0%)
EF2169 (EpaY)	467	Membrane protein, putative; 11 TMH, O-Antigen ligase (PF04932)		Polymerizes the decoration building blocks	O-antigen ligase family protein [*Lactobacillus paracasei*] Sequence ID: WP_123156247.1 (463 AA) 124/462(27%) 232/462(50%) 46/462(9%)
EF2168 (EpaZ)	274	LicD1 protein, putative; PF04991		Adds Rbo-P on a TA chain	LicD family protein [*Lactobacillus camelliae*] Sequence ID: WP_054665109.1 (274 AA) 143/274(52%) 185/274(67%) 3/274(1%)
EF2167	323	Glycosyl transferase, group 2 family protein; PF00535	**Predicted rhamnosyltransferase GBS1484** (315 AA) of *Streptococcus agalactiae* NEM316: 31% (75/244) amino acid identity and 53% (130/244) amino acid similarity (Sutcliffe et al, 2008).	Transfers Rha on a TA chain	Glycosyltransferase [*Lactobacillus cacaonum*] Sequence ID: WP_057829283.1 (308 AA) 108/292(37%) 158/292(54%) 22/292(7%)
EF2166	473	Membrane protein, putative; 12 TMH		Transports the decoration chain to the external face of the cytoplasmic membrane	Hypothetical protein [*Virgibacillus halodenitrificans*] Sequence ID: WP_077358726.1 (472 AA) 214/474(45%) 317/474(66%) 3/474(0%)
EF2165	324	NAD-dependent epimerase/dehydratase family protein; PF01370	**UDP-N-acetylglucosamine 4-epimerase WbpP** (341 AA) of *Pseudomonas aeruginosa* 36% (116/321) amino acid identity and 55% (178/321) amino acid similarity (Ishiyama et al, 2004).	Converts UDP-GlcNac to UDP-GalNac	NAD-dependent epimerase/dehydratase family protein [*Lactococcus lactis*] Sequence ID: WP_032946528.1 (309 AA) 199/312(64%) 246/312(78%) 6/312(1%)
EF2164	603	Membrane protein, putative; 11 TMH, PF11808		Links the TA chains to form the decoration building block	Hypothetical protein [*Kineothrix alysoides*] Sequence ID: WP_031392404.1 (455 AA) 144/457(32%) 245/457(53%) 29/457(6%); Hypothetical protein [Lactobacillus composti] Sequence ID: WP_035452870.1 (460 AA) 135/441(31%) 232/441(52%) 22/441(4%); hypothetical protein [Lactobacillus selangorensis] Sequence ID: WP_057771220.1 (454 AA) 128/437(29%) 219/437(50%) 16/437(3%); hypothetical protein [Lactobacillus sakei] Sequence ID: WP_076648356.1 (425 AA) 126/423(30%) 211/423(49%) 12/423(2%)

[a]: TMH for trans membrane helice(s)

[b]: with the exception of the functionality of EF2167 homolog, which is predicte

c: refrences cited:

Soldo B, Lazarevic V, Karamata D. 2002. tagO is involved in the synthesis of all anionic cell-wall polymers in *Bacillus subtilis* 168. Microbiology 148:2079-87.

Steiner K, Novotny R, Werz DB, Zarschler K, Seeberger PH, Hofinger A, Kosma P, Schaffer C, Messner P. 2008. Molecular basis of S-layer glycoprotein glycan biosynthesis in *Geobacillus stearothermophilus*. J Biol Chem 283:21120-33.

Sanchez-Rodriguez A, Tytgat HL, Winderickx J, Vanderleyden J, Lebeer S, Marchal K. 2014. A network-based approach to identify substrate classes of bacterial glycosyltransferases. BMC Genomics 15:349.

James DB, Gupta K, Hauser JR, Yother J. 2013. Biochemical activities of *Streptococcus pneumoniae* serotype 2 capsular glycosyltransferases and significance of suppressor mutations affecting the initiating glycosyltransferase Cps2E. J Bacteriol 195:5469-78.

Rush JS, Edgar RJ, Deng P, Chen J, Zhu H, van Sorge NM, Morris AJ, Korotkov KV, Korotkova N. 2017. The molecular mechanism of N-acetylglucosamine side-chain attachment to the Lancefield group A carbohydrate in *Streptococcus pyogenes*. J Biol Chem 292:19441-19457.

Steiner K, Novotny R, Patel K, Vinogradov E, Whitfield C, Valvano MA, Messner P, Schaffer C. 2007. Functional characterization of the initiation enzyme of S-layer glycoprotein glycan biosynthesis in *Geobacillus stearothermophilus* NRS 2004/3a. J Bacteriol 189:2590-8.

Brockhausen I, Hu B, Liu B, Lau K, Szarek WA, Wang L, Feng L. 2008. Characterization of two beta-1,3-glucosyltransferases from *Escherichia coli serotypes* O56 and O152. J Bacteriol 190:4922-32.

Baur S, Marles-Wright J, Buckenmaier S, Lewis RJ, Vollmer W. 2009. Synthesis of CDP-activated ribitol for teichoic acid precursors in *Streptococcus pneumoniae*. J Bacteriol 191:1200-10.

Gao Y, Liu B, Strum S, Schutzbach JS, Druzhinina TN, Utkina NS, Torgov VI, Danilov LL, Veselovsky VV, Vlahakis JZ, Szarek WA, Wang L, Brockhausen I. 2012. Biochemical characterization of WbdN, a beta1,3-glucosyltransferase involved in O-antigen synthesis in enterohemorrhagic *Escherichia coli* O157. Glycobiology 22:1092-102.
